# Identification of the GABARAP binding determinant in PI4K2A

**DOI:** 10.1042/BSR20240200

**Published:** 2024-10-23

**Authors:** Yan Chen, Barbara Barylko, John P. Eichorst, Joachim D. Mueller, Joseph P. Albanesi

**Affiliations:** 1 School of Physics and Astronomy, University of Minnesota, Minneapolis, MN 55455, U.S.A.; 2 Department of Pharmacology, University of Texas Southwestern Medical Center, Dallas, TX 75390, U.S.A.

**Keywords:** ATG8, fluorescence fluctuation spectroscopy, GABARAP, LIR motif, phosphatidylinositol 4-kinase 2A

## Abstract

GABARAP is a member of the ATG8 family of ubiquitin-like autophagy related proteins. It was initially discovered as a facilitator of GABA-A receptor translocation to the plasma membrane and has since been shown to promote the intracellular transport of a variety of other proteins under non-autophagic conditions. We and others have shown that GABARAP interacts with the Type II phosphatidylinositol 4-kinase, PI4K2A, and that this interaction is important for autophagosome-lysosome fusion. Here, we identify a 7-amino acid segment within the PI4K2A catalytic domain that contains the GABARAP interaction motif (GIM). This segment resides in an exposed loop that is not conserved in the other mammalian Type II PI 4-kinase, PI4K2B, explaining the specificity of GABARAP binding to the PI4K2A isoform. Mutation of the PI4K2A GIM inhibits GABARAP binding and PI4K2A-mediated recruitment of cytosolic GABARAP to subcellular organelles. We further show that GABARAP binds to mono-phosphorylated phosphoinositides, PI3P, PI4P, and PI5P, raising the possibility that these lipids contribute to the binding energies that drive GABARAP–protein interactions on membranes.

## Introduction

Phosphatidylinositol 4-phosphate (PI4P) is the major substrate in the synthesis of key signaling lipids, phosphatidylinositol (4,5)-bisphosphate (PI(4,5)P_2_) and phosphatidylinositol (3,4,5)-trisphosphate (PIP_3_), but also functions independently in a variety of endomembrane trafficking pathways, including Golgi-derived vesicle formation, endolysosomal transport, and autophagy [1–3]. In mammalian cells, PI4P is generated by two families of phosphatidylinositol 4-kinase (PI4Ks), Types II and III, each family containing two members (PI4K2A and 2B; PI4K3A and 3B) [4]. PI4K2A has been implicated in a variety of intracellular membrane trafficking pathways, including Golgi budding, endosomal recycling, and both endolysosomal and autolysosomal degradation [5–7]. For example, we reported that PI4K2A generates a pool of PI4P that is critical for autophagosome–lysosome fusion 8,9], perhaps by facilitating the recruitment of the scaffold protein ATG14 (autophagy-related 14) to autophagosomes [10]. In addition, PI4K2A-generated PI4P contributes to late-stage autophagy by enhancing the association of the SNARE syntaxin 17 (STX17) with autophagosomes [11] and by recruiting lipid transfer proteins for the formation of endoplasmic reticulum-autolysosome contact sites [12]. Another report also established the importance of PI4P for the recruitment of STX17 to autophagosomes [13], but the source of PI4P was not identified in that study. As shown by Balla’s group [14], PI4K2A also generates a pool of PI4P on late endosomes that is phosphorylated by PIP5Kγ to synthesize PI(4,5)P_2_. The acute conversion of PI4P to PI(4,5)P_2_ results in the dissociation of Rab7 from late endosomes and the consequent release of PLEKHM1 (pleckstrin homology and RUN domain containing M1), a GABARAP-binding protein implicated in autophagosome-lysosome fusion.

Upon induction of autophagy, recruitment of PI4K2A to mature autophagosomes requires the intervention of GABARAP (GABA-A receptor-associated protein) [8], which had previously been shown to interact with PI4K2A [15,16]. GABARAP is a member of the ATG8 (autophagy-related protein 8) family of ubiquitin-like proteins that participate in multiple steps of the autophagosome life cycle. The ATG8 family is divided into the LC3 (LC3A, LC3B, and LC3C) and the GABARAP (GABARAP, GABARAPL-1, and GATE-16) subfamilies, all of which are anchored into membranes via conjugation of their C-terminal glycines to phosphatidylethanolamine (PE). Although GABARAP has been most extensively characterized for its roles in autophagy [17], it was initially characterized as an intracellular trafficking factor for the GABA-A receptor [18–20]. Since then, it was shown to facilitate transport of the transferrin receptor [21], the kappa-opioid receptor (KOR) [22], the angiotensin II Type 1A receptor (AT_1_R) [23], the Na-dependent P_i_-cotransporter IIa (NaP_i_-IIa) [24], the transient receptor potential vanilloid (TRPV1) [25], and HIV-1 Nef [26]. GABARAP has also been reported to promote the transport of the N-cadherin/β-catenin complex from the endoplasmic reticulum (ER) to the Golgi [27]. Recently, GABARAP was found to interact directly with EGF receptors and to enhance their surface expression by shunting them to the recycling endocytic pathways [28]. Lipidation of GABARAP is not required for its promotion of AT_1_R translocation [29] but is important for its binding to and translocation of the GABA-A receptor [30] and KOR [31].

We previously reported that in live cells under non-autophagic conditions, EGFP-GABARAP is recruited to small (50–100 nm) cytoplasmic vesicles by co-expressed mCherry-PI4K2A [32]. We also showed that GABARAP lipidation was not essential for this recruitment. In the present study, we localize the GABARAP interaction motif in PI4K2A to an exposed loop within its catalytic domain.

## Materials and methods

### Materials

The pEGFP-C1, the mCherry-C1, and pEGFP-N1 vectors were purchased from Clontech (Mountainview, CA). Transfection reagent: TransFectin was from Bio-Rad (Hercules, CA) and Lipofectamine 3000 was from ThermoFisher Scientific (Waltham, MA). Anti-PI4K2A antibodies, generated in our laboratory, were raised against a synthetic peptide comprising residues 2-17 of PI4K2A and affinity purified by absorption against peptides attached to nitrocellulose filters. Glutathione resin was from Roche (Basel, Switzerland). [γ-^32^P]-ATP was from ThermoFisher. Unless otherwise mentioned, all other reagents were purchased from Sigma-Aldrich (St. Louis, MO).

### Plasmid construction

GABARAP was amplified from YFP-GABARAP (a gift from Zvulun Elazar (Weizmann Institute of Science, Rehovot, Israel) by using a 5′ primer that encodes a SacII restriction site and a 3′ primer that encodes an BamHI site and was ligated into pEGFP-C1 and mCherry-C1 backbone. The PI4K2A clone was prepared from a rat brain cDNA library as previously described [33] and was ligated into pEGFP-C1.

### Cell culture and transfection

HeLa and U2OS cells were obtained from American Type Culture Collection (ATCC, Manassas, VA) and maintained in 10% fetal bovine serum (JR Scientific, Woodland, CA) and DMEM media. All cells were cultured using standard sterile technique and grown in a humidified 5% CO_2_ atmosphere at 37°C until the cells reached approximately 95% confluence. Transient transfections of cDNA constructs were performed using TransFectin according to the manufacturer’s instructions. Fluorescence measurements were carried out 24 h after transfection. Growth medium was replaced with L-15 Medium (Leibovitz) in 10% fetal bovine serum immediately before measurement. For kinase activity assays, HeLa cells were grown on 10 cm plates and transfected when they reached approximately 90% confluency using Lipofectamine 3000. Assays were performed 20 h after transfection.

### Heterospecies partition analysis (HSP)

Dual-color fluorescence fluctuation measurements were performed on a modified Zeiss Axiovert 200 microscope (Thornwood, NY) using a Ti:sapphire laser (Tsunami, Spectra-Physics, Mountain View, CA) to achieve two-photon excitation. Fluorescence fluctuation data were collected for 60 s using a 63× Plan Apochromat oil immersion objective (N.A. = 1.4). An excitation wavelength of 1000 nm was chosen to simultaneously excite EGFP and mCherry for dual-color fluorescence experiments. The excitation power was kept close to 1 mW at 1000 nm, which is sufficiently low to avoid saturation. The fluorescence emission was split by color with a 580 nm dichroic mirror (585DCXR, Chroma Technology, Rockingham, VT) into two distinct detection channels. To eliminate the reflected fluorescence of mCherry into the green channel, an appropriate band pass filter (FF01-510/84-25 Semrock, Rochester, NY) was added to the beam path. The collected fluorescence intensity data of both channels were analyzed using the dual color hetero-species partition (HSP) algorithm as previously described [34]. The HSP algorithm identifies the HSP-brightness vector **b** = (*b*_r_, *b*_g_) of the heterospecies, which represents the fluorescence brightness of EGFP-labeled particles as well as any associated mCherry contributions in the red and green detection channel, respectively. Each HSP-brightness is displayed as a point in a two-axis brightness plot. If the heterospecies contains only the green EGFP species, the *b*_g_ and *b*_r_ ratio will follow the ratio of EGFP intensity split of the two detection channels. In this case, the **b** vector will fall along the ‘green species only line’. If the heterospecies values consist of particles carrying both EGFP and mCherry labeled proteins, the ratio of *b*_g_ and *b*_r_ will move to the right of the ‘green species only line’ in the brightness plot, which is termed the ‘green-red co-mobile zone’.

### Purification of GABARAP

For bacterial production GST-GABARAP was made using EGFP-GABARAP as a template. GABARAP cDNA sequence was introduced into the pGST.parallel 1 vector (gift from Hong Zhang, UTSW Medical Center) with a TEV cleavage site (ENLYFQ) at the N-terminus. Protein was expressed in *Escherichia coli* Rosetta 2 cells. Cells were harvested after growing for 22 h at 16°C in the presence of 0.5 mM isopropyl ß-D-1-thiogalactopyranoside (IPTG). Transformed cells were resuspended in buffer containing 20 mM HEPES (pH 8.0), 100 mM NaCl, 1 mM reducing agent tris(2-carboxyethyl)phosphine (TCEP), 0.2 mM PMSF, and a protease inhibitor cocktail comprising 10 mg/liter each of N a-p-tosyl-L-lysine chloromethyl ester, N a-p-tosyl-L-arginine methyl ester, N a-p-tosylL-lysine chloromethyl ketone, leupeptin, and pepstatin A (Buffer A) containing lysozyme (0.05 mg/ml; ∼3 nM). The cell suspension was sonicated and centrifuged at 100,000 × ***g*** for 30 min at 4°C. The extract was incubated with glutathione resin in buffer A. The resin was washed first with buffer A containing 0.2% Triton X-100, then with buffer A containing 1 M NaCl (without detergent). The bound protein was eluted with 50 mM glutathione. Glutathione was removed by dialysis against buffer containing 20 mM HEPES, pH 7.5, 100 mM NaCl, 1 mM TCEP, and 0.2 mM PMSF and aliquots of purified protein were frozen in liquid N_2_. GST was provided by Dr. Melanie Cobb (UT Southwestern).

### Lipid binding analysis

Binding of GABARAP to lipids was determined using flotation and protein-lipid overlay (‘PIP-strip’) assays. For flotation assays, liposomes were prepared from a porcine brain total lipid extract [35] (Avanti Polar Lipids, Inc., CAS:86088-88-2, containing (wt/wt) 9.6% PC, 16.7% PE, 10.6% PS, 2.8% PA, 1.6% PI, 58.7% unknown) supplemented with 1% rhodamine-labeled PE (Avanti Polar Lipids, Inc., Alabaster, AL) to facilitate identification of sucrose layers. Aliquots containing 2.5 mg lipids were dried under a stream of nitrogen followed by overnight drying under vacuum. Dried lipids were resuspended in 1 ml buffer containing 20 mM HEPES (pH 7.4) and 100 mM NaCl, followed by 10 freeze/thaw cycles in liquid nitrogen and bath sonication. To remove aggregated lipids, liposomes were extruded 10 times through 0.2 μm filters using a Mini–Extruder (Avanti Polar Lipids). Flotation assays were carried out according to Senju et al. [36]. Purified GST-tagged GABARAP (5 μM) or GST alone (as negative control) was mixed with liposomes (50 μl for 100 μl reaction), incubated for 1 h at room temperature, and mixed with an equal volume of 60% sucrose solution prepared in 20 mM HEPES, pH 7.5 and 100 mM NaCl. Immediately prior to incubation with liposomes, protein solutions were centrifuged for 1 h at 265,000 × ***g*** at 25°C to remove potential aggregates. Lipid-protein mixtures (100 μl in 30% sucrose) were then loaded on the bottom of ultracentrifugation tubes, overlayed with 150 μl of buffer containing 25% sucrose solution followed by 50 μl buffer without sucrose. The samples were ultracentrifuged in a Beckmann TL-100 centrifuge at 25°C for 1 h at 214,000 × ***g*** using a TLS-55 rotor. Fractions (40 μl) were collected from the top of the centrifuge tubes. Samples were subjected to SDS-PAGE, gels were stained with Coomassie blue, and images of the gels were analyzed using the LI-COR Odyssey system. For the protein-lipid overlay method, PIP strips (Echelon Biosciences, Salt Lake City, Utah) were blotted with 3% lipid-free BSA in phosphate-buffered saline (PBS) for 2 h at room temperature, followed by overlay with GST-GABARAP (∼25 nM) or GST (∼38 nM) in 2% BSA in PBS overnight at 4°C, then immunoblotted with anti-GST antibody.

### Preparation of membrane fractions

HeLa cells transfected with untagged or N-terminally EGFP-tagged PI4K2A (wild-type (WT) and mutant) were washed with ice cold PBS and scraped from Petri dishes in a solution containing 0.25 M sucrose, 20 mM Tris-HCl (pH 7.5), 100 mM NaCl, 1 mM EDTA, protease inhibitors and phosphatase inhibitors. Cells were lysed by two freeze–thaw cycles and passage through a 27-gauge needle. The lysates were centrifuged at 1,000 × ***g*** for 5 min to remove cell debris and nuclei. The supernatant (post-nuclear supernatant) was then centrifuged at 200,000 × ***g*** for 15 min to separate cytosol from membranes. The resulting membrane pellets were resuspended in lysis buffer and centrifuged again as above. The procedure was repeated three times to remove soluble phosphatidylinositol kinases. The final pellets were homogenized in lysis buffer and used for measuring kinase activity. Assays were performed in the absence and presence of Triton X-100, which stimulates PI 4-kinases but inhibits PI 3-kinases.

### Lipid kinase assay

PI4K activity was determined by measuring phosphorylation of endogenous phosphatidylinositol (PI) using [γ-^32^P]-ATP (10 mCi/ml) as radioactive phosphate donor [33]. The assays were performed either without detergent or in the presence of 0.5% Triton X-100 at 26°C for 30 min. Lipids were extracted according to Bligh and Dyer [37] and separated by thin-layer chromatography (TLC) in a solvent system consisting of n-propyl alcohol/H_2_O/NH_4_OH (65:20:15). As the major product was PI4P, in most experiments PI4P production was measured directly by scintillation counting without resolving the lipids on TLC. Activities of the expressed kinase were estimated by subtracting the activity in membranes obtained from mock-transfected cells. The relative amounts of kinase in samples were estimated by quantitative immunoblotting using anti-PI4K2A antibody and fluorescently-labeled secondary antibody in the LI-COR Odyssey system. Only experiments with similar expression levels were used to calculate the activities of the kinase. Therefore, different amounts of cells were used to prepare membrane fractions for the assays.

### Other methods

Protein concentration was determined using the modified Lowry method according to Peterson [38] with BSA as a standard. SDS-PAGE was carried out according to Laemmli [39]. For immunoblotting, proteins were transferred to nitrocellulose and immunoblotted with the indicated antibodies. Bound primary antibodies were detected and quantified using fluorescently labeled secondary antibody in the LI-COR Odyssey system.

## Results

### Identification of the GABARAP binding determinant in PI4K2A

**Figure 1:**
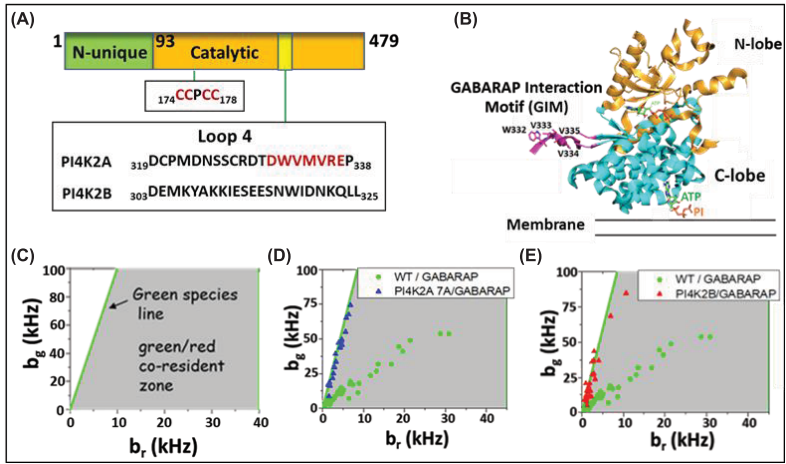
Identification of the GABARAP binding motif in PI4K2A (**A**) Scheme of Type II PI 4-kinases highlighting the location of the diverse Loop 4 sequences within the highly conserved catalytic domains of PI4K2A and PI4K2B. Mutation of residues marked in red inhibit GABARAP binding. (**B**) Orientation of the GABARAP binding motif with respect to the ATP and membrane binding sites in PI4K2A. (**C–E**) Heterospecies partition (HSP) analysis showing comigration of GABARAP with PI 4-kinases. (C) HSP concept. (D) Comigration of GABARAP with WT PI4K2A (green dots) but not with the 7A mutant in which residues 330-336 were replaced with alanines (blue triangles). (E) Comigration of GABARAP with WT PI4K2A (green dots) but not with PI4K2B (red triangles). Note that 30 of the 47 data points for PI4K2A^WT^ in panels (D) and (E) were reproduced with permission from the ACS from Chen et al., Comobility of GABARAP and phosphatidylinositol 4-Kinase 2A on cytoplasmic vesicles (2018) Biochemistry 57: 3556-3559.

Most proteins that bind ATG8 family members contain an LC3-interacting region (LIR) motif. The consensus sequence is [W/F/Y]-X-X-[L/I/V] [40]. According to an LIR prediction program [41], rat PI4K2A has three high scoring potential LIR motifs: _109_EFEVVV_114_, _222_NYSAID_227_, and _331_DWVMVRE_337_. All three of these motifs are located within the catalytic core of the kinase (approximately residues 93-478 in the rat PI4K2A sequence [42]; Figure 1A), consistent with our finding that a truncated PI4K2A mutant comprising residues 93-397 binds to GABARAP [8]. A subset of LIR motifs, defined by the sequence [W/F]-[V/I]-X-V, has been designated the GABARAP Interaction Motif (GIM) due to its preference of binding to GABARAP over LC3 family members [43]. Because PI4K2A selectively binds to GABARAP over LC3, we hypothesized that the third predicted LIR motif, which conforms to the GIM sequence, is the key GABARAP binding site in PI4K2A (Figure 1B) and generated a mutant, PI4K2A^7A^ in which amino acids _330_DWVMVRE_336_ are converted to _330_AAAAAAA_336_.

To determine if the 7A mutation disrupts the association of PI4K2A with GABARAP, we employed a variant of fluorescence fluctuation spectroscopy (FFS) known as dual-color heterospecies partition (HSP) analysis (see Materials and Methods). Dual-color HSP analysis allows detection and quantification of hetero-interactions in cells. HSP filters out red-labeled proteins that do not interact with green-labeled proteins and determines the brightness in the green and red channel, thereby specifying the average oligomeric composition of the interacting species. The data are displayed as HSP brightness vectors. Vesicles containing only EGFP-tagged proteins would express brightness vectors with a specific ratio in their green (*b*_g_) and red (*b*_r_) channel brightness indicated by the green line in Figure 1C. We previously used dual-color HSP analysis to demonstrate that in cells co-expressing EGFP-PI4K2A and mCherry-GABARAP, HSP brightness vectors for EGFP-PI4K2A were distributed within the green/red co-resident zone, indicating that PI4K2A and GABARAP co-migrate on the same cytoplasmic vesicles. To establish consistency between previous and current results, Figure 1D combines 30 data points from our previous publication [32], reproduced with permission from the ACS, and 17 new data points. In contrast, all the HSP brightness vectors for EGFP-PI4K2A^7A^ fell on the green line (Figure 1D), demonstrating that PI4K2A^7A^ does not recruit mCherry-GABARAP to vesicles.

**Figure 2:**
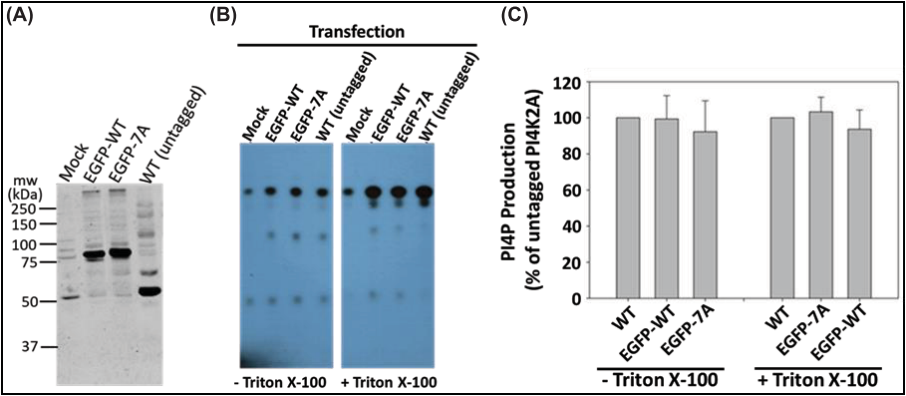
Lack of effect of mutating the GABARAP binding motif on the enzymatic activity of PI4K2A (**A**) Immunoblots of membrane fractions from HeLa cells expressing WT EGFP-PI4K2A (EGFP-WT), EGFP-PI4K2A^7A^ (EGFP-7A) or untagged WT PI4K2A (WT (untagged)). Membranes were blotted with anti-PI4K2A. (**B**) Thin layer chromatography (TLC) showing PI4P production in membranes isolated from transfected HeLa cells. Assays were performed in the presence or absence of Triton X-100, which stimulates PI 4-kinases but inhibits PI 3-kinases. PI4P production is increased equally by expression of WT and mutant kinases, and EGFP-tagging does not diminish activity. (**C**) Quantification of TLC assays based on four separate experiments.

Mammalian cells express a second Type II PI 4-kinase family member, PI4K2B, which differs primarily from PI4K2A in the non-catalytic N-terminal domain. Except for an exposed loop (loop 4; Figure 1A) the catalytic domains of the two kinases are highly conserved (68%/81% sequence identity/similarity). PI4K2B contains the first two predicted LIR motifs but not the GIM, which is located in loop 4. As shown in Figure 1E, EGFP-PI4K2B failed to recruit GABARAP to cytoplasmic microvesicles. To determine if the 7A mutation affects the lipid kinase activity of PI4K2A, we assayed PI4P production of membranes from HeLa cells expressing untagged PI4K2A^WT^, EGFP-PI4K2A^WT^, or EGFP-PI4K2A^7A^. The 7A mutation had no significant effect on the lipid kinase activity of PI4K2A (Figure 2), as expected based on the distance between the GIM-containing loop and the PI4K2A catalytic site.

**Figure 3:**
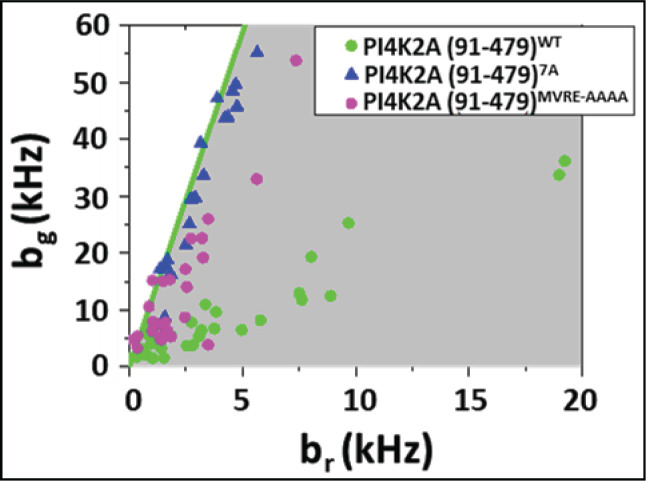
HSP analysis of cells expressing EGFP-tagged PI4K2A catalytic domains (residues 91-478) and mCherry-tagged GABARAP Results show comigration of GABARAP with WT PI4K2A (91-478) (green dots) but not with (91-478) containing the 7A mutation (blue triangles). A PI4K2A (91-478) construct in which residues _333_MVRE_336_ within the GABARAP binding motif are mutated to alanine shows reduced association with PI4K2A (purple dots).

To further define the GABARAP binding site in PI4K2A, we mutated four residues within the seven-residue GIM-containing motif in the context of the PI4K2A catalytic domain (residues 91-478). We had previously reported that a PI4K2A deletion mutant (PI4K2A-(91-478)) lacking the N-terminal disordered region co-immunoprecipitates with GABARAP [8]. Using HSP analysis, we confirmed that EGFP-PI4K2A-(91-478)^WT^ (Figure 3, green dots), but not EGFP-PI4K2A-(91-478)^7A^ (Figure 3, blue triangles) co-migrates with mCherry-GABARAP on cytoplasmic vesicles in live cells, confirming that the N-terminal, so-called ‘regulatory’ domain does not contribute to binding. We then mutated four residues (_334_MVRE_337_) within the seven-residue GABARAP binding motif in PI4K2A-(91-478) and found that this mutation strongly suppressed, although did not abrogate, association with PI4K2A (Figure 3, purple dots). Thus, mutation of the C-terminal valine in the [W/F]-[V/I]-X-V GIM motif was sufficient to severely disrupt the PI4K2A–GABARAP interaction.

### Identification of PI4K2A binding determinants in GABARAP

**Figure 4:**
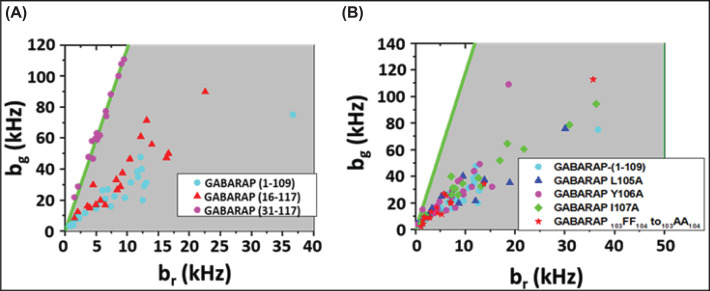
HSP analysis of cells expressing EGFP-PI4K2A and mCherry-GABARAP mutants (**A**) Analysis of the association of GABARAP truncation mutants lacking residues 110-117 (turquoise dots), residues 1-15 (red triangles), or residues 1-30 (purple dots) with PI4K2A. (**B**) Analysis of the effect of point mutations within a C-terminal segment of GABARAP on its association with PI4K2A. Data showing association of GABARAP (1-109) (turquoise dots) are reproduced from panel (A) for comparison.

GABARAP contains an N-terminal microtubule-binding domain (residues 1-26) [44] and a C-terminal ubiquitin-like domain (residues 27-117). Like other ATG8 proteins, GABARAP binds to LIR/GIM domains through two hydrophobic binding pockets [45]. Most residues that comprise these binding pockets reside in the ubiquitin-like domain, although residues within the N-terminal domain (e.g., K24 and Y25) have been shown to contribute to the interaction of GABARAP with Unc5-like-kinase 1 (ULK1) [46]. It is likely that the N-terminal domain of GABARAP is also involved in PI4K2A binding, as a deletion mutant lacking the N-terminal 30 amino acids (GABARAP-(31-117)) fails to associate with PI4K2A-containing vesicles (Figure 4A, purple dots). Deletion of residues 1-15 did not prevent the interaction (Figure 4, red triangles).

Upon induction of autophagy, GABARAP and other ATG8 family members undergo C-terminal lipidation, which stabilizes their association with membranes. This modification involves ATG4-mediated C-terminal proteolysis, which exposes a glycine residue that is conjugated to the amino headgroup of PE. Although the reaction is catalyzed by activation of several ATG proteins, including E1-like ATG7, E2-like ATG3, and the E3-like ATG12–ATG5–ATG16 complex, it can also occur under non-autophagic conditions, albeit at very low levels. We previously reported that C-terminal lipidation of GABARAP is not required for its association with PI4K2A, as unlipidated GABARAP^1-115^ co-migrates with PI4K2A-containing vesicles [32]. In the same study, we found that GABARAP^1-102^ does not co-migrate with these vesicles. Following up on this observation, we tested the effect of deleting residues _110_SDESVYGL_117_ from GABARAP. As shown in Figure 4 (turquoise dots), mCherry-GABARAP^1-109^ co-migrates with EGFP-PI4K2A, suggesting that critical PI4K2A binding determinants reside with segment _103_FFLYIA_108_. To determine if individual residues within this segment contribute to PI4K2A binding, we introduced the following mutations into full-length GABARAP: _103_FF_104_ to _103_AA_104_, L105A, Y106A, and I107A. None of these mutations prevented GABARAP from associating with PI4K2A-containing vesicles, as detected by HSP analysis (Figure 4B), suggesting that multiple residues within segment 103-108 must be mutated simultaneously to suppress the GABARAP–PI4K2A interaction.

### Binding of unlipidated GABARAP to mono-phosphorylated phosphoinositides

**Figure 5:**
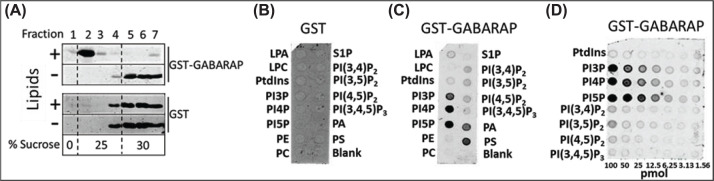
Binding of GABARAP to lipids (**A**) Flotation assay showing the binding of GST-GABARAP, but not GST, to liposomes that float to the 0–25% sucrose interface after centrifugation. Seven fractions were collected from the top to the bottom of the gradient, samples were electrophoresed, and gels were stained with Coomassie blue. The figure represents the result of one of three separate experiments. Percentages of liposome-bound GST-GABARAP in the three experiments were 100%, 88.3%, and 95.8%, as determined using the LI-COR imaging system. Uncropped gels are shown in Supplementary Figure S1. (**B–D**) Determination of the binding of GST (B) and GST-GABARAP (C) to different lipids and of GST-GABARAP to different amounts of phosphoinositides (D) by ‘PIP-Strip’ overlay. Membranes blotted with designated lipids were overlaid with ∼25 nM GST-GABARAP or ∼38 nM GST (as a negative control) and then immunoblotted with anti-GST antibody.

All six ATG8 orthologs, including GABARAP, bind directly to cardiolipin-containing liposomes, independently of their conjugation to PE [47,48]. Addition of ceramide enhances the binding, which was analyzed using flotation assays [48]. Using similar assays, we have confirmed that bacteriallyexpressed (and hence unlipidated) GABARAP binds to liposomes composed of a brain lipid mixture [35] (Figure 5A). We then used a lipid-protein overlay assay (‘PIP strip’) to test whether GABARAP displays any selectivity for binding to phosphoinositides. As shown in Figure 5B, GABARAP preferentially interacts with phosphatidylinositol monophosphates (PI3P, PI4P, and PI5P), with only slight binding to phosphatidylinositol polyphosphates (PI(3,4)P_2_, PI(4,5)P_2_, PI(3,5)P_2_, and PI(3,4,5)P_3_). Interestingly, the ATG8 family member LC3 binds to ceramides, which may facilitate its role in mitophagy, but fails to interact with PI4P or polyphosphoinositides [47]. Although the assay indicated a slightly higher affinity for PI5P over PI4P and PI3P (Figure 5C), the latter two lipids are present in much higher abundance on Golgi and endosomal membranes, the likely sources of PI4K2A-containing vesicles.

## Discussion

Following up on prior observations that PI4K2A interacts with GABARAP [8,15,32] and that the two proteins cooperate in promoting autophagosome-lysosome fusion [8,9], we have now localized the GABARAP interaction motif to a 7-amino acid segment within the catalytic domain of PI4K2A. Mutation of these seven residues to alanines abrogates the recruitment of cytosolic GABARAP to vesicles by co-expressed PI4K2A. Identification of a minimal GABARAP binding-deficient PI4K2A mutant could provide a relatively selective tool with which to investigate the cellular functions of the PI4K2A–GABARAP interaction. For example, PI4K2A^7A^ may fail to rescue processes requiring PI4K2A–GABARAP interactions in PI4K2A-depleted cells.

As in our previous publication [32], we used FFS as our primary approach to investigate the association of GABARAP with PI4K2A in live cells. By virtue of its palmitoylation, PI4K2A behaves as an integral membrane protein and stably associates with cellular organelles and cytoplasmic vesicles. The mobility of PI4K2A is much lower on organelle membranes than on vesicles. Thus, the organelle-associated pool of PI4K2A contributes little to the intensity fluctuations measured in FFS experiments and can be avoided by carefully choosing the position of two-photon laser excitation beam. We used this strategy in our FFS measurements to preferentially detect and record fluctuations from the mobile pool of PI4K2A that is associated with small, rapidly moving vesicles, and then to analyze these fluctuations using the HSP approach.

**Figure 6:**
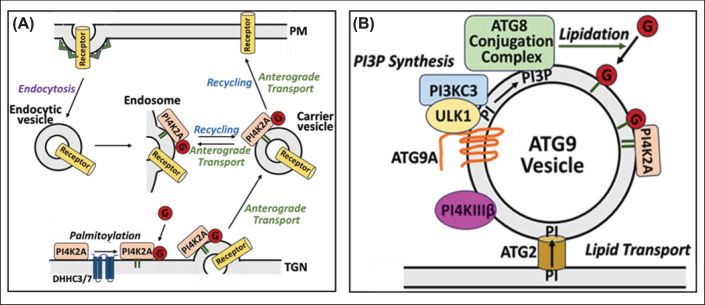
Hypothetical models showing association of PI4K2A with GABARAP (**A**) Non-autophagic trafficking. Palmitoylated PI4K2A recruits unlipidated, cytosolic GABARAP (G) to Golgi and/or endosomal membranes. These membranes are the source of carrier vesicles that transport cargo (e.g., receptors) anterogradely from the trans-Golgi network (TGN) or recycling endosomes to the plasma membrane (PM). (**B**) Autophagic trafficking. ATG9A-containing vesicles dock on autophagosome initiation sites on the endoplasmic reticulum and expand into mature autophagosomes by ATG2-mediated lipid transport and ATG9A-mediated lipid scrambling. ULK1/PI3KC3-mediated PI3P production on the docked vesicles results in the recruitment of the ATG8 conjugation complex, which lipidates GABARAP, thereby anchoring it into the nascent autophagosome membrane. ATG9A vesicles carry both PI4K3B (PI4KIIIβ) and PI4K2A, which produce pools of PI4P utilized in autophagosome formation and autophagosome-lysosome-fusion, respectively. According to our model, lipidated PI4K2A and GABARAP are stably associated throughout the autophagosomal process. See text for more details.

Although our studies were performed under nutrient-rich non-autophagic conditions, it is safe to assume that the same GABARAP binding motif is required for the GABARAP–PI4K2A interaction on autophagosomes. At present, non-autophagic transport processes that involve both GABARAP and PI4K2A have not been identified. However, GABARAP was reported to bind directly to EGF receptors and shunt them to the recycling endocytic pathways [28], in which PI4K2A has also been implicated [49,50]. According to our hypothetical model (Figure 6A), unlipidated GABARAP is recruited from the cytosol to Golgi and/or endosomal membranes via direct binding to PI4K2A, which had undergone palmitoylation by the palmitoyl acyltransferases DHHC3 and DHHC7 on the Golgi [51]. We propose that the PI4K2A- and GABARAP-containing particles detected in our FFS experiments represent carrier vesicles that emerge from these membranes and deliver cargo (such as EGF receptors) anterogradely to the plasma membrane.

Under nutrient-deprived conditions, PI4K2A-derived PI4P has an independent function in autophagosome-lysosome fusion, perhaps by promoting the recruitment of ATG14 [10] and/or STX17 [11] to mature autophagosomes, and by providing substrate for the production of PI(4,5)P_2_ on late endosomes [14]. Autophagosomes form by extension and sealing of crescent-shaped double-membrane structures known as isolation membranes or phagophores, which are docked on specific regions of the endoplasmic reticulum (ER) [52]. The membrane source for phagophore initiation and expansion is still subject to debate, but there is general agreement that 50–100 nm vesicles containing ATG9A (Atg9 in yeast), the only transmembrane ATG protein, are involved [53,54]. Indeed, a recently proposed model suggests that phagophores originate from a single ATG9A-containing vesicle that buds from the Golgi and/or recycling endosomes and docks on the ER [55,56]. This vesicle is believed to expand via ATG2-mediated lipid transfer from the ER, followed by the ‘scrambling’ of lipids between the two leaflets of the bilayer by ATG9A itself. Of the four PI4K isoforms, only PI4K2A and PI4K3B have been implicated in autophagy in mammalian cells [7]. Both kinases travel to the site of autophagosome formation on ATG9A-containing vesicles [57,58]. PI4K3B, which does not bind to GABARAP [8], has a role in autophagosome formation [57], whereas PI4K2A functions specifically in late-stage autophagy. According to our hypothetical model (Figure 6B), GABARAP undergoes lipidation on ATG9A vesicles by the ATG8 conjugation complex, which is itself recruited to ATG9A vesicles by a pool of PI3P generated by phosphatidylinositol 3-kinase C3-Complex 1 (PI3KC3-C1) activated by ULK1. Palmitoylated PI4K2A encounters lipidated GABARAP on these vesicles, forming a stable association that lasts until autophagosome-lysosome fusion.

As part of this investigation, we found that GABARAP interacts with negatively charged phospholipids, including mono-phosphorylated (but not polyphosphorylated) phosphoinositides. Although we have not yet identified the binding determinant for anionic lipids in GABARAP, we note that its N-terminal region, and that of other ATG8 family members, contains a positively charged motif which, in GABARAP, binds to the acidic C-terminal region of tubulin [18,59]. A recent report indicates that the N-terminal domains of lipidated ATG8 family members interact directly with the same membrane leaflets in which the C-terminal PE moieties are embedded [60]. The same study showed that residues within the N-terminal domain of GABARAP penetrate the lipid bilayer, thereby contributing to autophagosome membrane expansion. Our finding raises the possibility that mono-phosphorylated phosphoinositides contribute to the binding energy that drives GABARAP–protein interactions on membranes. For example, PI4P may enhance the binding of GABARAP to PI4K2A on endosomes and mature autophagosomes and PI3P may contribute to its recruitment to phagophore membranes upon induction of autophagy, when PI3KC3-C1 is activated. We also note that PI5P, which showed the strongest binding to GABARAP, has been implicated in autophagosome biogenesis [61].

## Conclusion

GABARAP plays an essential role in the recruitment to autophagosomes of PI4K2A, which generates a pool of PI4P that contributes to autophagosome-lysosome fusion. In this study, we have identified critical binding determinants within the two proteins. The GABARAP interaction motif (GIM) comprises a 7-amino acid segment in a disordered loop within the PI4K2A catalytic domain. PI4K2A binding determinants in GABARAP are distributed between its N-terminal domain (residues 1-26) and a short C-terminal segment (residues 103-108). We also confirmed that GABARAP associates with artificial phospholipid vesicles and demonstrated a preferential binding to monophosphorylated phosphoinositides, PI3P, PI4P, and PI5P. This work provides tools for future investigations of the role of the PI4K2A–GABARAP interaction in late stages of autophagy and in cargo trafficking under non-autophagic conditions.

## Supplementary Material

Supplementary Figure S1

## Data Availability

All materials, data and associated protocols will be made available upon request.

## Open Access

Open access for this article was enabled by the participation of University of Minnesota in an all-inclusive *Read & Publish* agreement with Portland Press and the Biochemical Society under a transformative agreement with Individual.

## Accession Codes

Rat PI4K2A: Q99M64.1, Human PI4K2B: Q8TCG2, Human GABARAP: O95166.

## Abbreviations

DMEM: Dulbecco’s modified Eagle’s medium
EGFP: enhanced green fluorescent protein
GABA: γ-aminobutyric acid
GABARAP: GABA_A_ receptor-associated protein
GATE-16: Golgi-associated ATPase enhancer of 16 kDa
GST: glutathione-S-transferase
HEPES: (4-(2-hydroxyethyl)-1-piperazineethanesulfonic acid)
LC3: microtubule-associated protein light chain 3
PAGE: polyacrylamide gel electrophoresis
SDS: sodium dodecyl sulfate
TEV protease: Tobacco etch virus protease
YFP: yellow fluorescent protein
