# Supplementary material for: Identification of the GABARAP binding determinant in PI4K2A
Source: Biosci Rep. 2024 Oct 23;44(10):BSR20240200. doi: 10.1042/BSR20240200 (PMC11499380; doi:10.1042/BSR20240200)
Supplement: Supplementary Figure S1 [file BSR-2024-0200_supp.pdf]

Supplementary Figure S1

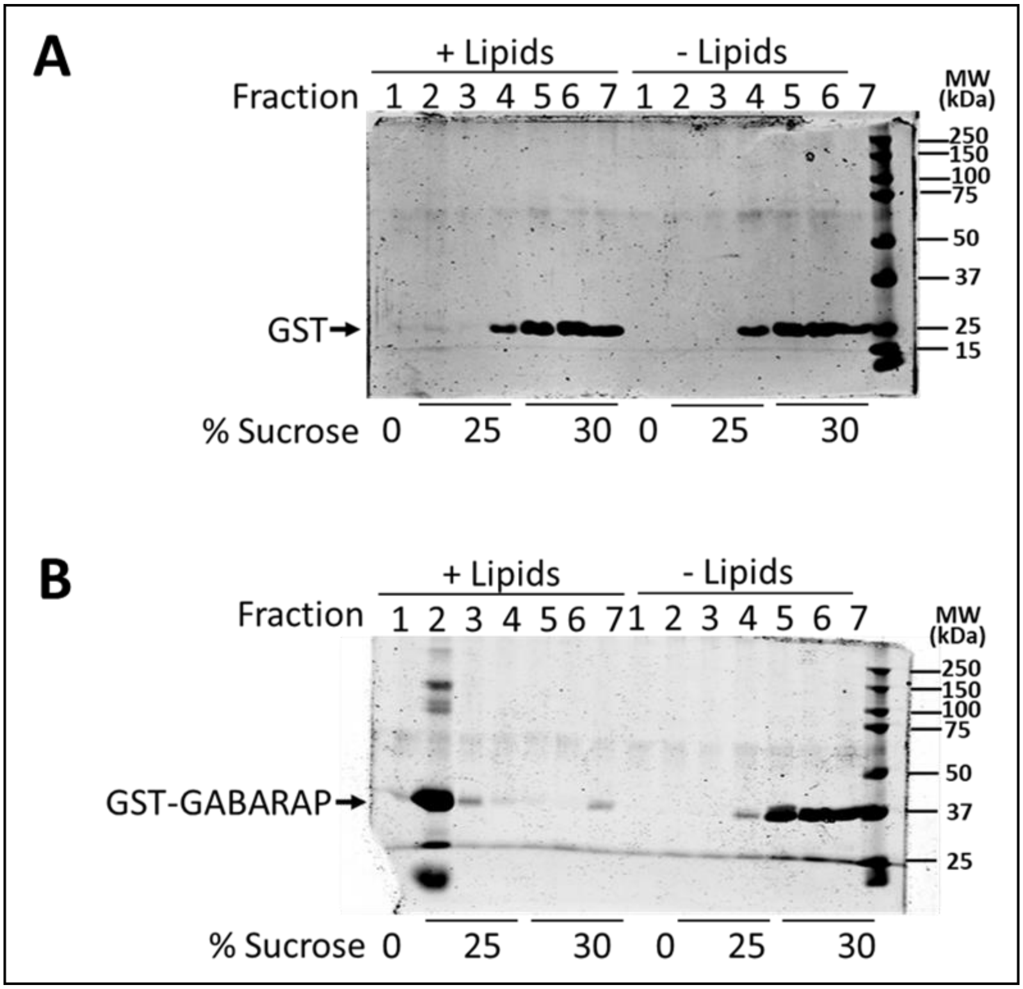

Uncropped gels corresponding to Figure 5A. Flotation assay showed the binding of GST-GABARAP (panel B), but not of GST alone (panel A) to liposomes that float to the 0-25% sucrose interface after centrifugation. Seven fractions were collected from the top to the bottom of the gradient, samples were electrophoresed, and gels were stained with Coomassie blue.
